# Supplementary material for: Factors driving choices between types and brands of influenza vaccines in general practice in Austria, Italy, Spain and the UK
Source: PLoS One. 2021 Jun 15;16(6):e0252836. doi: 10.1371/journal.pone.0252836 (PMC8205138; doi:10.1371/journal.pone.0252836)
Supplement: S1 File — (PDF) [file pone.0252836.s001.pdf]

Understanding influenza vaccine use and influenza vaccine type use in practice – a GP survey (English version)

General Information

This survey is about influenza vaccination and the choice of vaccine types for different risk groups.

This survey is conducted by DRIVE, a consortium that aims to annually assess influenza vaccine effectiveness in Europe. For more information on DRIVE please visit www.drive-eu.org.

Results of the survey will be analysed at country-level and used to support the interpretation of influenza vaccine effectiveness results.

1. Background

Practice identifier used in DRIVE data

Years of practice

Specialization (if any)

Understanding influenza vaccine use and influenza vaccine type use in practice – a GP survey (English version)

PART 1 – Questions to understand who is offered influenza vaccination

This survey is about which groups of patients are offered influenza vaccination and how.

2. For which patient populations do you provide influenza vaccination? (check all that apply)

- ☐ Children
- ☐ Adults under 65y
- ☐ Pregnant women
- ☐ Adults 65+y
- ☐ Institutionalized patients

3. Are any of the above populations overrepresented at your practice? (check all that apply)

- ☐ No
- ☐ Yes, children
- ☐ Yes, adults under 65y
- ☐ Yes, pregnant women
- ☐ Yes, Adults 65+y
- ☐ Yes, institutionalized patients

4. How is seasonal influenza vaccine offered to patients that are part of the vaccine recommendations? (check all that apply)

- [ ] All recommended patients are contacted (e.g. through a letter)
- [ ] A subset of recommended patients is contacted (e.g. through a letter) (please describe subset in the next question)
- [ ] All recommended patients are proactively offered influenza vaccination if they present for a consultation during the vaccination campaign or influenza season
- [ ] A subset of recommended patients is proactively offered influenza vaccination if they present for a consultation during the vaccination campaign or influenza season (please describe subset in the next question)
- [ ] Influenza vaccination is only offered upon request from the patient
- [ ] Other (please specify)

5. Please describe which patients are part of the subset (if applicable)

6. Any comments on how influenza vaccination is offered to patients?

7. To what extent do you agree with the following statement: Patients who perceive themselves to be at higher risk are more likely to accept vaccination

- ○ Fully agree
- ○ Mostly agree
- ○ Mostly disagree
- ○ Fully disagree

8. To what extent do you agree with the following statement: Patients who are more health conscious are more likely to accept vaccination

- ◯ Fully agree
- ◯ Mostly agree
- ◯ Mostly disagree
- ◯ Fully disagree

9. Any comments on other factors that make your patients more likely to accept vaccination?

PART 2 - Choice of vaccine type and brand

Survey questions for England

This part of the survey is about the choice of vaccine types, and choice of vaccine brand among those that are vaccinated.

By vaccine types we mean groups of vaccines, such as: conventional trivalent influenza vaccines (TIV), egg-based quadrivalent influenza vaccines (egg-based QIV, such as Vaxigrip Tetra, Influvac Tetra, Fluarix Tetra), cell-based QIV (Flucelvax Tetra), adjuvanted trivalent vaccines (aTIV, Fluad), and high dose trivalent vaccine.

By vaccine brands we mean vaccines of the same type, for example Vaxigrip Tetra and Influvac Tetra are two different vaccine brands of the same type.

10. Do you have patients aged 65+y under your care?

- ○ Yes
- ○ No

PART 2 - Choice of vaccine type and brand

National flu immunisation programme 2019/20:
For those aged 65 and over, there are three vaccines that JCVI advised are equally suitable for use. The adjuvanted trivalent influenza vaccine (adjuvanted TIV) continues to be recommended for this age group as it is likely to be a more effective vaccine than the standard dose non-adjuvanted trivalent and egg-based quadrivalent influenza vaccines. Equally suitable, is the newly licenced cell-based quadrivalent influenza vaccine (cell-based QIV). Whilst also suitable for this age group, the newly licenced high dose trivalent influenza vaccine (TIV-HD) is not eligible for reimbursement under the NHS flu vaccination programme because it has a significantly higher list price.

11. How many vaccines are available at your practice/pharmacy for patients aged 65+ years?

- ◯ Only one of the following: adjuvanted TIV, cell-based QIV, TIV-HD, egg-based QIV, non-adjuvanted TIV
- ◯ Two or more of the following: adjuvanted TIV, cell-based QIV, TIV-HD, egg-based QIV, non-adjuvanted TIV

12. Please indicate which vaccines are available at your practice/pharmacy for patients 65+ years? (check all that apply)

- ☐ Adjuvanted TIV
- ☐ Cell-based QIV
- ☐ TIV-HD
- ☐ Egg-based QIV
- ☐ Non-adjuvanted TIV

PART 2 - Choice of vaccine type and brand

13. Does patient preference for one of the vaccines influence your choice of vaccine type?

- Yes
- No

14. Does willingness of patient to pay for a non-reimbursed vaccine (i.e. TIV-HD) influence your choice of vaccine type?

- Yes
- No
- Not applicable

15. Does price influence your choice of vaccine type for an individual patient?

- Yes
- No

If yes, please comment on price

16. Does vaccine availability drive your choice of vaccine type?

- Yes
- No

Please comment on vaccine availability

17. Do individual patient characteristics (except contraindication) influence your choice of vaccine type?

- ◯ Yes
- ◯ No

PART 2 - Choice of vaccine type and brand

18. Which patient characteristics influence your choice of vaccine type? (check all that apply)

- [ ] Younger age
- [ ] Older age
- [ ] Presence of multiple comorbidities
- [ ] Presence of specific comorbidities (please specify below)
- [ ] Higher severity of comorbidities
- [ ] Frailty
- [ ] Other (please specify)

19. Please comment on how this affects your choice of vaccine type

Vaccine type in adults 18-64y at increased risk from flu because of a long term health condition

20. Do you have adults aged 18-64y at increased risk from flu because of a long-term health condition under your care?

- ◯ Yes
- ◯ No

Vaccine type in adults 18-64y at increased risk from flu because of a long term health condition

National flu immunisation programme 2019/20:
For those aged 18 to 64 years, there are two vaccines which JCVI advises are equally suitable for use. The standard egg-grown quadrivalent influenza vaccine (egg-based QIV) and the newly licenced cell-based quadrivalent influenza vaccine (cell-based QIV).

21. Two QIV vaccine types are equally recommended, cell-based and egg-based. Which of these are available at your practice/pharmacy for patients aged 18-64 years?

- ◯ Cell-based QIV
- ◯ Egg-based QIV
- ◯ Both

Vaccine type in adults 18-64y at increased risk from flu because of a long term health condition

22. Does patient preference for cell-based QIV or egg-based QIV influence your choice of vaccine type?

- Yes
- No

23. Does price influence your choice of vaccine type for an individual patient?

- Yes
- No

If yes, please comment on price

24. Does vaccine availability drive your choice of vaccine type?

- Yes
- No

Please comment on vaccine availability

25. Do individual patient characteristics (except contraindication) influence your choice of vaccine type?

- Yes
- No

Vaccine type in adults 18-64y at increased risk from flu because of a long term health condition

26. Which patient characteristics influence your choice of vaccine type? (check all that apply)

- ☐ Younger age
- ☐ Older age
- ☐ Presence of multiple comorbidities
- ☐ Presence of specific comorbidities (please specify below)
- ☐ Higher severity of comorbidities
- ☐ Frailty
- ☐ Profession
- ☐ Other (please specify)

27. Please comment on how this affects your choice of vaccine type

Vaccine brands

28. Multiple brands of egg-based QIV vaccines are licensed for use in children aged 6 months to 2 years (from manufacturers GSK, Masta, Sanofi Pasteur) and in adults (from manufacturers GSK, Masta, Mylan, Sanofi Pasteur). Do you have more than one egg-based QIV vaccine brand available for your patients?

- ◯ Yes
- ◯ No

29. Do you perceive these vaccines as clinically interchangeable?

- ◯ Yes, fully
- ◯ Yes, mostly
- ◯ No, mostly not
- ◯ No, not at all

Please comment your answer

DRIVE

Development of Robust and Innovative Vaccine Effectiveness

imi innovative medicines initiative

efpia

Understanding influenza vaccine use and influenza vaccine type use in practice – a GP survey (German version)

Allgemeine informationen

Bei dieser Umfrage geht es um die Grippeimpfung und die Wahl der Impfstofftypen für verschiedene Risikogruppen.

Diese Umfrage wird von DRIVE durchgeführt, einem Konsortium, das die Wirksamkeit von Grippeimpfstoffen in Europa jährlich bewerten wird. Weitere Informationen zu DRIVE finden Sie unter www.drive-eu.org.

Die Ergebnisse der Erhebung werden auf Länderebene analysiert und zur Interpretation der Wirksamkeitsergebnisse von Grippeimpfstoffen verwendet. Die Umfrage ist anonym und wir bitten Sie lediglich ihr Bundesland anzugeben. Um jedoch einen Überblick über die Rücklaufquote und die Teilnehmer dieser Umfrage zu erhalten, können sie gerne Ihre Initialen neben dem Bundesland angeben, dies ist selbstverständlich optional.

1. Allgemeines

Bundesland (Initialen des Arztes, optional)

Praxiserfahrung in Jahren

Spezialisierung

TEIL 1 – Fragen zum Thema wem eine Schutzimpfung gegen Influenza (Grippe) angeboten wird

2. Für welche Patientengruppen bieten Sie eine Grippeimpfung an? (Zutreffendes ankreuzen)

- ☐ Kinder
- ☐ Erwachsene (<65 Jahre)
- ☐ Schwangere Frauen
- ☐ Erwachsene (65+ Jahre)
- ☐ Institutionalisierte Patienten (Personen in Pflegeheimen, Altersheimen, etc.)

3. Sind eine oder mehrere Patientengruppen überrepräsentiert in Ihre Praxis?(Zutreffendes ankreuzen)

- ☐ Nein
- ☐ Ja, Kinder
- ☐ Ja, Erwachsene (<65 Jahre)
- ☐ Ja, Schwangere Frauen
- ☐ Ja, Erwachsene (65+ Jahre)
- ☐ Ja, Institutionalisierte Patienten (Personen in Pflegeheimen, Altersheimen, etc.)

4. Wie wird die saisonale Grippeimpfung Patienten angeboten, die Teil der Risikogruppen für Impfempfehlungen sind? (Zutreffendes ankreuzen)

- [ ] Alle Risikopatienten werden (z.B. per Post) kontaktiert und eine Grippeimpfung wird angeboten
- [ ] Ein Teil der Risikopatienten wird kontaktiert und eine Grippeimpfung wird angeboten (z.B. per Post) (Bitte beschreiben Sie die Teilmenge in der nächsten Frage)
- [ ] Allen Risikopatienten, die während der Impfkampagne oder der Grippesaison die Praxis aufsuchen, wird proaktiv eine Grippeimpfung angeboten
- [ ] Einem Teil der Risikopatienten, die während der Impfkampagne oder der Grippesaison die Praxis aufsuchen, wird proaktiv eine Grippeimpfung angeboten (Bitte beschreiben Sie die Teilmenge in der nächsten Frage)
- [ ] Grippeimpfungen werden nur auf Anfrage des Patienten angeboten
- [ ] Sonstiges (bitte angeben)

5. Bitte beschreiben Sie, welchem Teil der Patienten eine Grippeimpfung angeboten wird (falls zutreffend)

6. Sonstige Kommentare dazu, wie den Patienten eine Grippeimpfung angeboten wird?

7. Inwieweit stimmen Sie der folgenden Aussage zu: Risikopatienten akzeptieren eher eine Impfung

- ( ) Stimme zu
- ( ) Stimme eher zu
- ( ) Stimme eher nicht zu
- ( ) Stimme nicht zu

8. Inwieweit stimmen Sie der folgenden Aussage zu: Gesundheitsbewusste Patienten akzeptieren eher eine Impfung

- ○ Stimme zu
- ○ Stimme eher zu
- ○ Stimme eher nicht zu
- ○ Stimme nicht zu

9. Sonstige Kommentare zu anderen Faktoren, die die Wahrscheinlichkeit erhöhen, dass Ihre Patienten eine Impfung akzeptieren?

TEIL 2 – Wahl des Impfstofftyps und der Marke

Österreich
In dieser Umfrage geht es um die Wahl des Impfstofftyps und der Marke.

Mit Impfstofftypen sind Gruppen von Impfstoffen gemeint, wie beispielsweise: inaktivierte Vierfachimpfstoffe (QIV) (welche eibasiert oder zellbasiert sein können), adjuvantierte (wirkverstärkte) Dreifachimpfstoffe (aTIV).

Mit Impfstoffmarken sind spezifische Impfstoffprodukte gemeint, zum Beispiel Vaxigrip Tetra oder Fluad. Für einige Impfstofftypen gibt es mehrere Impfstoffmarken.

10. Haben Sie Patienten im Alter von 9 bis 17 Jahren?

- ◯ Ja
- ◯ Nein

Impftstofftyp für Kinder (9-17 Jahre)

Impfplan Österreich 2019

	Trivalente adjuvantierte Influenza-Vakzine	Tetravalente, inaktivierte Influenza-Vakzine	Tetravalente Lebend-Vakzine[1]
Vollendetes 6. Lebensmonat bis vollendetes 2. Lebensjahr		+[2]	
Vollendetes 2. bis vollendetes 18. Lebensjahr		+[2]	+[2]

[2] Bei der erstmaligen Impfung von Kindern bis zum vollendeten 9. Lebensjahr (tetravalenter Totimpfstoff) oder wenn das Kind bisher erst eine einzige Impfung erhalten hat (abweichend von der Fachinformation), sollen 2 Impfungen im Abstand von mindestens 4 Wochen gegeben werden.

11. In Österreich sind zwei quadrivalente Influenza-Impfstofftypen (QIV) erhältlich, zellbasiert (Flucelvax Tetra) und eibasiert (Fluarix Tetra, Influvac Tetra, Vaxigrip Tetra). Welche Impfstoffe gibt es in Ihrer Praxis/Apotheke für Kinder von 9 bis 17 Jahren ?

- ◯ Nur zellbasierter QIV
- ◯ Nur eibasierter QIV
- ◯ Beide

Impftstofftyp für Kinder (9-17 Jahre)

12. Hat die Bevorzugung von quadrivalentem Influenza-Impfstoff auf Zellbasis (Flucelvax Tetra) oder auf Eibasis (Fluarix Tetra, Influvac Tetra, Vaxigrip Tetra) durch den Patienten Einfluss auf Ihre Wahl des Impfstofftyps?

- ◯ Ja
- ◯ Nein

13. Beeinflusst die Bereitschaft des Patienten für quadrivalenten Influenza-Impfstoff (QIV) (Flucelvax Tetra) oder eibasierten QIV (Fluarix Tetra, Influvac Tetra, Vaxigrip Tetra) zu zahlen Ihre Wahl des Impfstofftyps?

- ◯ Ja
- ◯ Nein

14. Beeinflusst der Preis Ihre Wahl des Impfstofftyps für einen einzelnen Patienten?

- ◯ Ja
- ◯ Nein

Wenn ja, bitte erläutern Sie den Einfluss des Impfstoffpreises.

15. Bestimmt die Impfstoffverfügbarkeit Ihre Wahl des Impfstofftyps?

- ◯ Ja
- ◯ Nein

Bitte erläutern Sie die Bedeutung der Impfstoffverfügbarkeit.

16. Beeinflussen Faktoren wie Alter, Grunderkrankungen, etc. eines einzelnen Patienten (außer Kontraindikationen) Ihre Wahl des Impfstofftyps bei Kindern im Alter von 9-17 Jahre?

- ◯ Ja
- ◯ Nein

Impftstofftyp für Kinder (9-17 Jahre)

17. Welche Patienteneigenschaften beeinflussen Ihre Wahl des Impfstofftyps? (Zutreffendes ankreuzen)

- [ ] Jüngeres Alter
- [ ] Höheres Alter
- [ ] Vorhandensein mehrerer Begleiterkrankungen
- [ ] Vorliegen spezifischer Begleiterkrankungen (bitte angeben)
- [ ] Höherer Schweregrad der Begleiterkrankungen
- [ ] Gebrechlichkeit

Sonstiges (bitte angeben)

18. Bitte erläutern Sie, wie sich dies auf Ihre Wahl des Impfstofftyps auswirkt.

Impftstofftyp für Erwachsene (18-64 Jahre)

19. Haben Sie Patienten im Alter von 18 bis 64 Jahren?

- ◯ Ja
- ◯ Nein

Understanding influenza vaccine use and influenza vaccine type use in practice – a GP survey (German version)

Impftstofftyp für Erwachsene (18-64 Jahre)

Impfplan Österreich 2019

	Trivalente adjuvantierte Influenza-Vakzine	Tetravalente, inaktivierte Influenza-Vakzine	Tetravalente Lebend-Vakzine[1]
Vollendetes 18. bis vollendetes 65. Lebensjahr		+	

20. In Österreich sind zwei quadrivalente Influenza-Impfstofftypen (QIV) erhältlich, zellbasiert (Flucelvax Tetra) und eibasiert (Fluarix Tetra, Influvac Tetra, Vaxigrip Tetra). Welche Impfstoffe gibt es in Ihrer Praxis/Apotheke für Erwachsene 18-64 Jahre?

- ◯ Nur zellbasierter QIV
- ◯ Nur eibasierter QIV
- ◯ Beide

Impftstofftyp für Erwachsene (18-64 Jahre)

21. Hat die Bevorzugung von quadrivalentem Influenza-Impfstoff auf Zellbasis (Flucelvax Tetra) oder auf Eibasis (Fluarix Tetra, Influvac Tetra, Vaxigrip Tetra) durch den Patienten Einfluss auf Ihre Wahl des Impfstofftyps?

- ○ Ja
- ○ Nein

22. Beeinflusst die Bereitschaft des Patienten, für quadrivalenten Influenza-Impfstoff (QIV) (Flucelvax Tetra) oder eibasierten QIV (Fluarix Tetra, Influvac Tetra, Vaxigrip Tetra) zu zahlen, Ihre Wahl des Impfstofftyps?

- ○ Ja
- ○ Nein

23. Beeinflusst der Preis Ihre Wahl des Impfstofftyps für einen einzelnen Patienten?

- ○ Ja
- ○ Nein

Wenn ja, bitte erläutern Sie den Einfluss des Impfstoffpreises.

24. Bestimmt die Impfstoffverfügbarkeit Ihre Wahl des Impfstofftyps?

- ◯ Ja
- ◯ Nein

Bitte erläutern Sie die Bedeutung der Impfstoffverfügbarkeit.

25. Beeinflussen Faktoren wie Alter, Grunderkrankungen, etc. eines einzelnen Patienten (außer Kontraindikationen) Ihre Wahl des Impfstofftyps bei Erwachsenen im Alter von 18-64 Jahre?

- ◯ Ja
- ◯ Nein

Impftstofftyp für Erwachsene (18-64 Jahre)

26. Welche Patienteneigenschaften beeinflussen Ihre Wahl des Impfstofftyps? (Zutreffendes ankreuzen)

- ☐ Jüngeres Alter
- ☐ Höheres Alter
- ☐ Vorhandensein mehrerer Begleiterkrankungen
- ☐ Vorliegen spezifischer Begleiterkrankungen (bitte angeben)
- ☐ Höherer Schweregrad der Begleiterkrankungen
- ☐ Gebrechlichkeit
- ☐ Beruf
- ☐ Sonstiges (bitte angeben)

27. Bitte erläutern Sie, wie sich dies auf Ihre Wahl des Impfstofftyps auswirkt.

Impftstofftyp für Risikopatienten (schwere chronische Erkrankung, schwere Immunsuppression)

28. Haben Sie Patienten, die wegen schwerer chronischer Erkrankung oder schwerer Immunsuppression als Grippe-Risikopatienten gelten?

- ◯ Ja
- ◯ Nein

Impftstofftyp für Risikopatienten (schwere chronische Erkrankung, schwere Immunsuppression)

Impfplan Österreich 2019

	Trivalente adjuvantierte Influenza-Vakzine	Tetravalente, inaktivierte Influenza-Vakzine	Tetravalente Lebend-Vakzine[1]
Risikopersonen (schwer chronisch Kranke, stark Immunsupprimierte)	+[4]	+[4]	

[4] Bei Immunsuppression sequentielle Impfung: zuerst trivalent adjuvantiert (teilweise altersabhängig auch off-label), im Intervall von mindestens 4 Wochen in Abhängigkeit von der epidemiologischen Lage eine weitere tri- oder tetravalent, inaktivierte, nicht adjuvantierte Impfung.

29. Welche Impfstoffe gibt es in Ihrer Praxis/Apotheke für Risikopatienten? (Zutreffendes ankreuzen)

- ◯ Nur trivalente, adjuvantierte Vakzine (aTIV; Fluad)
- ◯ Nur zellbasierter quadrivalenter Influenza-Impfstoff (QIV) (Flucelvax Tetra)
- ◯ Nur eibasierter QIV (Fluarix Tetra, Influvac Tetra, Vaxigrip Tetra)
- ◯ Sowohl aTIV als auch zellbasierter QIV
- ◯ Sowohl aTIV als auch eibasierter QIV
- ◯ Sowohl zellbasierter als auch eibasierter QIV
- ◯ Alle drei

Impftstofftyp für Risikopatienten (schwere chronische Erkrankung, schwere Immunsuppression)

30. Hat die Bevorzugung von trivalenter adjuvantierter Vakzine (Fluad), zellbasiertem quadrivalentem Influenza-Impfstoff (QIV) (Flucelvax Tetra) oder eibasiertem QIV (Fluarix Tetra, Influvac Tetra, Vaxigrip Tetra) durch den Patienten Einfluss auf Ihre Wahl des Impfstofftyps?

- ◯ Ja
- ◯ Nein

31. Beeinflusst die Bereitschaft des Patienten für trivalente adjuvantierte Vakzine (Fluad), zellbasierten quadrivalenten Influenza-Impfstoff (QIV) (Flucelvax Tetra) oder eibasierten QIV (Fluarix Tetra, Influvac Tetra, Vaxigrip Tetra) zu zahlen, Ihre Wahl des Impfstofftyps?

- ◯ Ja
- ◯ Nein

32. Beeinflusst der Preis Ihre Wahl des Impfstofftyps für einen einzelnen Patienten?

- ◯ Ja
- ◯ Nein

Wenn ja, bitte erläutern Sie den Einfluss des Impfstoffpreises.

33. Bestimmt die Impfstoffverfügbarkeit Ihre Wahl des Impfstofftyps?

- ◯ Ja
- ◯ Nein

Bitte erläutern Sie die Bedeutung der Impfstoffverfügbarkeit.

34. Beeinflussen Faktoren wie Alter, Grunderkrankungen, etc. eines einzelnen Patienten (außer Kontraindikationen) Ihre Wahl des Impfstofftyps bei Risikopatienten?

- ◯ Ja
- ◯ Nein

Impftstofftyp für Risikopatienten (schwere chronische Erkrankung, schwere Immunsuppression)

35. Welche patientenbezogenen Faktoren beeinflussen Ihre Wahl des Impfstofftyps? (Zutreffendes bitte ankreuzen)

- ☐ Jüngeres Alter
- ☐ Höheres Alter
- ☐ Vorhandensein mehrerer Begleiterkrankungen
- ☐ Vorliegen spezifischer Begleiterkrankungen (bitte angeben)
- ☐ Höherer Schweregrad der Begleiterkrankungen
- ☐ Gebrechlichkeit
- ☐ Sonstiges (bitte angeben)

36. Bitte erläutern Sie, wie sich dies auf Ihre Wahl des Impfstofftyps auswirkt.

Imfpstofftyp für Senioren (65+ Jahre)

37. Haben Sie Patienten im Alter von 65+ Jahren?

- ◯ Ja
- ◯ Nein

Imfpstofftyp für Senioren (65+ Jahre)

Impfplan Österreich 2019

	Trivalente adjuvantierte Influenza-Vakzine	Tetravalente, inaktivierte Influenza-Vakzine	Tetravalente Lebend-Vakzine[1]
Ab vollendetem 65. Lebensjahr	+	(+)[3]	

[3] Ist nach den epidemiologischen Daten mit hoher Wahrscheinlichkeit von einem intensiven Auftreten des Influenza B-Stammes, welcher nur durch die tetravalente Impfung abgedeckt wird, zu rechnen (B Yamagata), wird ergänzend oder von vornherein eine Impfung mit dem tetravalenten Impfstoff empfohlen.

38. Welche Impfstoffe gibt es in Ihrer Praxis/Apotheke für Senioren im Alter von 65+ Jahren? (Zutreffendes ankreuzen)

- ◯ Nur trivalente, adjuvantierte Vakzine (aTIV; Fluad)
- ◯ Nur zellbasierter quadrivalenter Influenza-Impfstoff (QIV) (Flucelvax Tetra)
- ◯ Nur eibasierter QIV (Fluarix Tetra, Influvac Tetra, Vaxigrip Tetra)
- ◯ Sowohl aTIV als auch zellbasierter QIV
- ◯ Sowohl aTIV als auch eibasierter QIV
- ◯ Sowohl zellbasierter als auch eibasierter QIV
- ◯ Alle drei

Imfpstofftyp für Senioren (65+ Jahre)

39. Hat die Bevorzugung von trivalenter adjuvantierter Vakzine (Fluad), zellbasiertem quadrivalentem Influenza-Impfstoff (QIV) (Flucelvax Tetra) oder eibasiertem QIV (Fluarix Tetra, Influvac Tetra, Vaxigrip Tetra) durch den Patienten Einfluss auf Ihre Wahl des Impfstofftyps?

- Ja
- Nein

40. Beeinflusst die Bereitschaft des Patienten für trivalente adjuvantierte Vakzine (Fluad), zellbasierten quadrivalenten Influenza-Impfstoff (QIV) (Flucelvax Tetra) oder eibasierten QIV (Fluarix Tetra, Influvac Tetra, Vaxigrip Tetra) zu zahlen Ihre Wahl des Impfstofftyps?

- Ja
- Nein

41. Beeinflusst der Preis Ihre Wahl des Impfstofftyps für einen einzelnen Patienten?

- Ja
- Nein

Wenn ja, bitte erläutern Sie den Einfluss des Impfstoffpreises.

42. Bestimmt die Impfstoffverfügbarkeit Ihre Wahl des Impfstofftyps?

- ◯ Ja
- ◯ Nein

Bitte erläutern Sie die Bedeutung der Impfstoffverfügbarkeit.

43. Beeinflussen Faktoren wie Alter, Grunderkrankungen, etc. eines einzelnen Patienten (außer Kontraindikationen) Ihre Wahl des Impfstofftyps bei Senioren im Altern von 65+ Jahren?

- ◯ Ja
- ◯ Nein

Impfstofftyp für Senioren (65+ Jahre)

44. Welche patientenbezogenen Faktoren beeinflussen Ihre Wahl des Impfstofftyps? (Zutreffendes bitte ankreuzen)

- ☐ Jüngeres Alter
- ☐ Höheres Alter
- ☐ Vorhandensein mehrerer Begleiterkrankungen
- ☐ Vorliegen spezifischer Begleiterkrankungen (bitte angeben)
- ☐ Höherer Schweregrad der Begleiterkrankungen
- ☐ Gebrechlichkeit
- ☐ Sonstiges (bitte angeben)

45. Bitte erläutern Sie, wie sich dies auf Ihre Wahl des Impfstofftyps auswirkt.

Impfstoffmarken (QIV)

46. Mehrere QIV Impfstoffmarken sind für die Verwendung zugelassen (z.B. Fluarix Tetra, Influvac Tetra, Vaxigrip Tetra). Haben Sie mehr als eine QIV Impfstoffmarke für Ihre Patienten zur Verfügung?

- ◯ Ja
- ◯ Nein

47. Sehen Sie diese Impfstoffe als klinisch gleichwertig/ austauschbar an?

- ◯ Ja
- ◯ Eher ja
- ◯ Eher nein
- ◯ Nein

Falls nicht oder eher nicht, geben Sie bitte an, warum Sie diese Impfstoffe nicht als klinisch gleichwertig/ austauschbar betrachten.

Understanding influenza vaccine use and influenza vaccine type use in practice – a GP survey (Italian version)

Introduzione alle domande del questionario

Questo sondaggio riguarda la vaccinazione antinfluenzale e la scelta dei tipi di vaccino per i diversi gruppi a rischio. Questo sondaggio è condotto da DRIVE, un consorzio che mira a valutare annualmente l'efficacia del vaccino antinfluenzale in Europa. Per ulteriori informazioni su DRIVE, potete visitare il sito www.drive-eu.org.

I risultati dell'indagine saranno interpretati a livello nazionale e verranno utilizzati a supporto dell'interpretazione dei risultati di efficacia del vaccino antinfluenzale.

1. Informazioni generali

Regione

Anzianità lavorativa (anni)

Specializzazioni (se ci sono)

2. Nome e cognome

NOTA BENE: l'inserimento del tuo nome e cognome è facoltativo e sarà usato solo per la finalità di collegare la tua risposta ai dati della sorveglianza virologica raccolti per il progetto DRIVE. Il tuo nome non sarà menzionato in alcun modo e non sarà presente nel dataset finale che sarà completamente anonimizzato.

Domande inerenti a chi viene offerta la vaccinazione antinfluenzale

Questo sondaggio riguarda i gruppi di pazienti a cui viene offerta la vaccinazione antiinfluenzale e la modalità di offerta.

3. A quali categorie di pazienti somministri il vaccino antinfluenzale? (indicarne una o più)

☐ Bambini che rientrano nei gruppi a rischio

☐ Adulti < 65 anni che rientrano nei gruppi a rischio

☐ Donne incinta

☐ Adulti 65+ anni che rientrano nei gruppi a rischio

☐ Pazienti istituzionalizzati

4. Alcune delle categorie di pazienti sopraelencate sono maggiormente rappresentate tra i tuoi assistiti? (indicarne una o più)

☐ No

☐ Sì, Bambini che rientrano nei gruppi a rischio

☐ Sì, Adulti < 65 anni che rientrano nei gruppi a rischio

☐ Sì, Donne incinta

☐ Sì, Adulti 65+ anni che rientrano nei gruppi a rischio

☐ Sì, Pazienti istituzionalizzati

5. In che modalità offri la vaccinazione antinfluenzale stagionale ai pazienti che appartengono alle categorie a rischio? (indicarne una o più)

- ☐ Tutti i pazienti che appartengono alle categorie a rischio sono contattati (e.s. invio lettera)
- ☐ Solo una parte di pazienti che appartengono alle categorie a rischio viene contattata (e.s. invio lettera) (Descrivi questo sottogruppo nella prossima domanda)
- ☐ Viene offerta la vaccinazione antinfluenzale in modo proattivo a tutti i pazienti che appartengono alle categorie a rischio solo se si presentano per un consulto medico durante la campagna vaccinale antinfluenzale
- ☐ La vaccinazione antinfluenzale è offerta in modo proattivo ad un sottogruppo di pazienti che appartengono alle categorie a rischio solo se si presentano per un consulto durante la campagna vaccinale antinfluenzale (Descrivi questo sottogruppo nella prossima domanda)
- ☐ La vaccinazione antinfluenzale è offerta ai soli pazienti che ne fanno richiesta
- ☐ Altro, prego specificare

6. Descrivi le categorie a rischio che contatti

7. Se vuoi commenta come viene offerta la vaccinazione antinfluenzale ai tuoi pazienti

8. In che misura sei d'accordo con la seguente affermazione: i pazienti che percepiscono di appartenere a una categoria a rischio sono maggiormente predisposti alla vaccinazione?

- ◯ Completamente d'accordo
- ◯ Parzialmente d'accordo
- ◯ Parzialemente in disaccordo
- ◯ Completamente in disaccordo

9. In che misura sei d’accordo con la seguente affermazione: i pazienti che sono più attenti alla loro salute sono maggiormente predisposti alla vaccinazione?

- ◯ Completamente d’accordo
- ◯ Parzialmente d’accordo
- ◯ Parzialmente in disaccordo
- ◯ Completamente in disaccordo

10. Se vuoi descrivi quali sono i fattori che influiscono sull’accettazione della vaccinazione

Scelta della tipologia vaccinale e relativa marca

Il seguente questionario riguarda la scelta della tipologia di vaccino e della relativa marca.

Per tipologia di vaccini intendiamo categorie come: il vaccino antinfluenzale quadrivalente inattivato (QIV) che può essere coltivato su uova o su colture cellulari.

Per marca vaccinale intendiamo specifici prodotti vaccinali, come per esempio il Vaxigrip Tetra, il Fluarix Tetra o il Flucelvax Tetra. Per queste tipologie vaccinali esistono più marchi commerciali.

Tipologia di vaccini nei soggetti di età compresa tra I 10 ed i 17 anni

11. Hai assisititi di età compresa tra i 10 ed i 17 anni?

- ○ Sì
- ○ No

Tipologie vaccinali in bambini 10-17 anni

Raccomandazioni per la stagione 2019-2020:
Considerato l’impatto dell'influenza B nei bambini e il potenziale di mis-match tra il ceppo circolante predominante dell’influenza B e il ceppo presente nel vaccino trivalente, l’uso della formulazione quadrivalente del vaccino antinfluenzale nei bambini e negli adolescenti sarebbe preferibile (QIV coltivato su uova o QIV coltivato su colture cellulari).

12. Quali tipologie di vaccini sono disponibili presso il tuo ambulatorio per la somministrazione ai soggetti di età compresa tra i 10 ed i 17 anni?

- ◯ Unicamente QIV coltivato su uova
- ◯ Unicamente QIV coltivato su colture cellulari
- ◯ Sia QIV coltivato su uova che QIV coltivato su colture cellulari

Tipologie vaccinali in bambini 10-17 anni

13. La preferenza dei genitori per una delle tipologie vaccinali condiziona la scelta del tipo di vaccino da somministrare ai bambini tra i 10 e i 17 anni?

- Sì
- No

14. La disponibilità del vaccino determina la scelta del tipo di vaccino da somministrare nei pazienti tra i 10 e i 17 anni?

- Sì
- No

Si prega di commentare la disponibilità del vaccino

15. Le caratteristiche dei singoli pazienti, come età, comorbilità, etc. (tranne la controindicazione), condizionano la scelta del tipo di vaccino da somministrare ai bambini di età compresa tra i 10 e i 17 anni?

- Sì
- No

Tipologie vaccinali in bambini 10-17 anni

16. Quali caratteristiche del paziente ti indirizzano verso la scelta della tipologia di vaccino? (indica tutte quelle pertinenti)

- ☐ Giovane età
- ☐ Età avanzata
- ☐ Presenza di comorbilità multiple
- ☐ Presenza di specifiche comorbilità (per favore specificare sotto)
- ☐ Maggiore gravità delle comorbilità
- ☐ Fragilità
- ☐ Altro, prego specificare

17. Commenta in che modo le caratteristiche indicate influiscono sulla scelta del tipo di vaccino (è più probabile che prescriva QIV coltivato su uova o coltivato su colture cellulari?)

Tipologie vaccinali in adulti 18-64 anni

18. Hai pazienti di età compresa tra i 18 e i 64 anni tra i tuoi assistiti?

- ◯ Sì
- ◯ No

Tipologie vaccinali in adulti 18-64 anni

Raccomandazioni per la stagione 2019-2020:
QIV coltivato su uova e QIV coltivato su colture cellulari sono i prodotti raccomandati per gli adulti con condizioni di malattia cronica e negli operatori sanitari.

19. Quali tipologie vaccini sono disponibili presso il tuo ambulatorio da somministrare ai pazienti di età compresa tra i 18 e i 64 anni?

- ◯ Unicamente QIV coltivato su uova
- ◯ Unicamente QIV coltivato su colture cellulari
- ◯ Sia QIV coltivato su uova che QIV coltivato su colture cellulari

Tipologie vaccinali in adulti 18-64 anni

20. La preferenza del paziente per una delle tipologie vaccinali condiziona la scelta del tipo di vaccino da somministrare agli adulti tra i 18 e i 64 anni?

- ◯ Sì
- ◯ No

21. La disponibilità del vaccino determina la scelta del tipo di vaccino da somministrare ai pazienti tra i 18 e i 64 anni?

- ◯ Sì
- ◯ No

Si prega di commentare la disponibilità del vaccino

22. Le caratteristiche dei singoli pazienti, come età, comorbilità, etc. (tranne la controindicazione), condizionano la scelta del tipo di vaccino da somministrare agli adulti di età compresa tra i 18 e i 64 anni?

- ◯ Sì
- ◯ No

Understanding influenza vaccine use and influenza vaccine type use in practice – a GP survey (Italian version)

Tipologie vaccinali in adulti 18-64 anni

23. Quali caratteristiche del paziente ti indirizzano verso la scelta della tipologia di vaccino? (indica tutte quelle pertinenti)

- ☐ Giovane età
- ☐ Età avanzata
- ☐ Presenza di comorbilità multiple
- ☐ Presenza di specifiche comorbilità (per favore specificare sotto)
- ☐ Maggiore gravità delle comorbilità
- ☐ Fragilità
- ☐ Professione
- ☐ Altro, prego specificare

24. Commenta in che modo le caratteristiche indicate influiscono sulla scelta del tipo di vaccino (è più probabile che prescriva QIV coltivato su uova o coltivato su colture cellulari?)

Understanding influenza vaccine use and influenza vaccine type use in practice – a GP survey (Italian version)

Tipologie vaccinali in adulti a partire dai 65 anni

25. Hai pazienti di età pari o superiore ai 65 anni tra i tuoi assistiti?

- ◯ Sì
- ◯ No

Understanding influenza vaccine use and influenza vaccine type use in practice – a GP survey (Italian version)

Tipologie vaccinali in adulti a partire dai 65 anni

Raccomandazioni per la stagione 2019-2020:
TIV adiuvato (Fluad), QIV coltivato su uova (Vaxigrip Tetra, Fluarix Tetra) e QIV (Flucelvax Tetra) coltivato su colture cellulari sono i prodotti raccomandati per gli adulti di età ≥65 anni.
Data l'importanza della malattia influenzale da virus A(H3N2) nei grandi anziani (75+) e l'evidenza di una migliore efficacia in questo gruppo di età, si prevede che, in questa categoria, la formulazione adiuvata del vaccino TIV, dovrebbe fornire una protezione superiore rispetto al vaccino non adiuvato e quadrivalente.

26. Quali tipologie di vaccini sono disponibili presso il tuo ambulatorio per la somministrazione ai pazienti di età pari o superiore a 65 anni?

- ◯ Unicamente QIV coltivato su uova
- ◯ Unicamente QIV coltivato su colture cellulari
- ◯ Unicamente TIV adiuvato
- ◯ Sia QIV coltivato su uova che su colture cellulari
- ◯ Sia QIV coltivato su uova che che TIV adiuvato
- ◯ Sia QIV coltivato su colture cellulari che TIV adiuvato
- ◯ Tutti e tre

Tipologie vaccinali in adulti a partire dai 65 anni

27. La preferenza del paziente per una delle tipologie vaccinali condiziona la scelta del tipo di vaccino da somministrare agli adulti di età superiore o uguale a 65 anni?

- ◯ Sì
- ◯ No

28. La disponibilità del vaccino determina la scelta del tipo di vaccino da somministrare agli adulti di età superiore o uguale a 65 anni?

- ◯ Sì
- ◯ No

Si prega di commentare la disponibilità del vaccino

29. Le caratteristiche dei singoli pazienti, come età, comorbilità, etc. (tranne la controindicazione), condizionano la scelta del tipo di vaccino da somministrare agli adulti di età superiore o uguale a 65 anni?

- ◯ Sì
- ◯ No

Tipologie vaccinali in adulti a partire dai 65 anni

30. Quali caratteristiche del paziente ti indirizzano verso la scelta della tipologia di vaccino? (indica tutte quelle pertinenti)

- ☐ Giovane età
- ☐ Età avanzata
- ☐ Presenza di più comorbilità
- ☐ Presenza di specifiche comorbilità (per favore specificare sotto)
- ☐ Maggiore gravità delle comorbilità
- ☐ Fragilità
- ☐ Altro, prego specificare

31. Commenta in che modo le caratteristiche indicate influiscono sulla scelta del tipo di vaccino (è più probabile che prescriva QIV coltivato su uova o coltivato su colture cellulari?)

Tipologie vaccinali in adulti a partire dai 65 anni

32. Utilizzi Fluad (invece del QIV coltivato su uova o del QIV coltivato su colture cellulari) nei pazienti tra i 65 e i 74 anni?

- ◯ Sì, sempre
- ◯ Sì, solitamente sì
- ◯ Qualche volta
- ◯ No, solitamente no
- ◯ No, mai

33. La preferenza del paziente per una delle tipologie vaccinali condiziona la scelta del tipo di vaccino da somministrare agli adulti tra i 65 e i 74 anni?

- ◯ Sì
- ◯ No

34. La disponibilità del vaccino determina la scelta del tipo di vaccino da somministrare agli adulti tra i 65 e i 74 anni?

- ◯ Sì
- ◯ No

Si prega di commentare la disponibilità del vaccino

35. Le caratteristiche dei singoli pazienti, come età, comorbilità, etc. (tranne la controindicazione), condizionano la scelta del tipo di vaccino da somministrare agli adulti di età compresa tra i 65 e i 74 anni?

- ◯ Sì
- ◯ No

Tipologie vaccinali in adulti a partire dai 65 anni

36. Quali caratteristiche del paziente ti indirizzano verso la scelta della tipologia di vaccino? (indica tutte quelle pertinenti)

- ☐ Giovane età
- ☐ Età avanzata
- ☐ Presenza di più comorbilità
- ☐ Presenza di specifiche comorbilità (per favore specificare sotto)
- ☐ Maggiore gravità delle comorbilità
- ☐ Fragilità
- ☐ Altro, prego specificare

37. Commenta in che modo le caratteristiche indicate influiscono sulla scelta del tipo di vaccino (maggiore probabilità di somministrare Fluad, QIV coltivato su uova o QIV coltivato su colture cellulari?)

Tipologie vaccinali in adulti a partire dai 65 anni

38. Somministri Fluad a chi ha 75 anni o più?

- Sì, sempre
- Sì, solitamente sì
- Qualche volta
- No, solitamente no
- No, mai

39. La preferenza del paziente per una delle tipologie vaccinali condiziona la scelta del tipo di vaccino da somministrare agli anziani di età superiore o uguale a 75 anni?

- Sì
- No

40. La disponibilità del vaccino determina la scelta del tipo di vaccino da somministrare agli anziani di età superiore o uguale a 75 anni?

- Sì
- No

Si prega di commentare la disponibilità del vaccino

41. Le caratteristiche dei singoli pazienti, come età, comorbilità, etc. (tranne la controindicazione), condizionano la scelta del tipo di vaccino da somministrare agli anziani di età superiore o uguale a 75 anni?

- ◯ Sì
- ◯ No

Tipologie vaccinali in adulti a partire dai 65 anni

42. Quali caratteristiche del paziente ti indirizzano verso la scelta della tipologia di vaccino? (indica tutte quelle pertinenti)

- [ ] Giovane età
- [ ] Età avanzata
- [ ] Presenza di più comorbilità
- [ ] Presenza di specifiche comorbilità (per favore specificare sotto)
- [ ] Maggiore gravità delle comorbilità
- [ ] Fragilità
- [ ] Altro, prego specificare

43. Commenta in che modo le caratteristiche indicate influiscono sulla scelta del tipo di vaccino (maggiore probabilità di somministrare Fluad, QIV coltivato su uova o QIV coltivato su colture cellulari?)

Marche vaccinali

44. In Italia sono disponibili più marche di vaccini QIV coltivati su uova (Vaxigrip Tetra, Fluarix Tetra, Influvac S Tetra). Hai più di una marca di vaccino QIV coltivato su uova disponibile per i tuoi pazienti?

- Sì
- No

45. Percepisci questi vaccini come clinicamente intercambiabili?

- Sì, completamente
- Sì, prevalentemente
- No, prevalentemente
- No, completamente

Se necessario, commenta la tua risposta

Understanding influenza vaccine use and influenza vaccine type use in practice – a GP survey (Spanish version)

Preguntas de la encuesta

Esta encuesta trata sobre la vacunación contra la influenza y la elección de los tipos de vacuna para diferentes grupos de riesgo.

DRIVE, un consorcio que tiene como objetivo evaluar anualmente la efectividad de la vacuna contra la gripe en Europa, realiza esta encuesta. Para obtener más información sobre DRIVE, visite www.drive-eu.org.

Los resultados de la encuesta se analizarán a nivel de país y se utilizarán para ayudar la interpretación de los resultados de efectividad de la vacuna contra la influenza.

La encuesta es voluntaria.
Los datos serán anonimizados y tratados de acuerdo con la Ley de Protección de Datos Personales.
Para resolver cualquier duda la persona de contacto es Cintia Muñoz Quiles (munoz_cin@gva.es).

1. General

Años de práctica ____________________

Especialidad (si hay) ____________________

Understanding influenza vaccine use and influenza vaccine type use in practice – a GP survey (Spanish version)

PARTE 1 - Preguntas para comprender a quién se le ofrece la vacuna contra la influenza

Esta encuesta trata sobre qué grupos de pacientes reciben la vacuna contra la influenza y cómo.

2. ¿Para qué poblaciones de pacientes recomienda la vacuna contra la gripe? (marque todo lo que corresponda)

- ☐ Niños
- ☐ Adultos menores de 65 años
- ☐ Mujeres embarazadas
- ☐ Adultos mayores de 65 años
- ☐ Pacientes institucionalizados

3. ¿Alguna de las poblaciones anteriores está sobrerrepresentada en su práctica? (marque todo lo que corresponda)

- ☐ No
- ☐ Sí, Niños
- ☐ Sí, Adultos menores de 65 años
- ☐ Sí, Mujeres embarazadas
- ☐ Sí, Adultos mayores de 65 años
- ☐ Sí, pacientes institucionalizados

4. ¿Cómo se ofrece la vacuna contra la influenza estacional a los pacientes que forman parte de las recomendaciones de la vacuna? (marque todo lo que corresponda)

- ☐ Todos los pacientes que forman parte de las recomendaciones de la vacuna se contactan (por ejemplo, a través de una carta)
- ☐ Se contacta a un subconjunto de pacientes que forman parte de las recomendaciones de la vacuna (por ejemplo, a través de una carta) (describa qué pacientes forman parte del subconjunto en la siguiente pregunta)
- ☐ A todos los pacientes que forman parte de las recomendaciones de la vacuna se les ofrece proactivamente la vacuna contra la influenza si se presentan para una consulta durante la campaña de vacunación o la temporada de influenza
- ☐ A un subconjunto de pacientes que forman parte de las recomendaciones de la vacuna se les ofrece proactivamente la vacuna contra la influenza si se presentan para una consulta durante la campaña de vacunación o la temporada de influenza (describa qué pacientes forman parte del subconjunto en la siguiente pregunta)
- ☐ La vacuna contra la influenza solo se ofrece a pedido del paciente
- ☐ Otro, por favor especifique

5. Describa qué pacientes forman parte del subconjunto (si corresponde)

6. ¿Algún comentario sobre cómo se ofrece la vacuna contra la influenza a los pacientes?

7. ¿En qué medida está de acuerdo con la siguiente declaración: los pacientes que se perciben a sí mismos como de mayor riesgo tienen más probabilidades de aceptar la vacunación?

- ○ Completamente de acuerdo
- ○ Mayormente de acuerdo
- ○ Mayormente en desacuerdo
- ○ Totalmente en desacuerdo

8. ¿En qué medida está de acuerdo con la siguiente declaración: los pacientes que son más conscientes de la salud tienen más probabilidades de aceptar la vacunación?

- Completamente de acuerdo
- Mayormente de acuerdo
- Mayormente en desacuerdo
- Totalmente en desacuerdo

9. ¿Algún comentario sobre otros factores que hacen que sus pacientes sean más propensos a aceptar la vacuna?

Understanding influenza vaccine use and influenza vaccine type use in practice – a GP survey (Spanish version)

PARTE 2 - Preguntas de la encuesta para España – Valencia

Esta parte de la encuesta trata sobre la elección de los tipos de vacunas.

Por tipos de vacuna nos referimos a grupos de vacunas, tales como: vacunas cuadrivalentes contra la influenza cultivada en huevo (QIV cultivada en huevo, como Vaxigrip Tetra), QIV cultivada en células (Flucelvax Tetra) y vacunas trivalentes adyuvadas (aTIV, Chiromas).

10. ¿Tiene pacientes de 65 años o más?

- ◯ Si
- ◯ No

PARTE 2 - Preguntas de la encuesta para España – Valencia

Recomendaciones de vacunación 2019-2020:

Se recomiendan las siguientes vacunas:

- Para grupos de riesgo de 6 meses a 9 años: Vaxigrip Tetra (vacuna cuadrivalente contra la gripe, cultivada en huevo)

- Para grupos de riesgo de 9 a 64 años: Flucelvax Tetra (vacuna cuadrivalente contra la influenza, cultivada en células)

- Para los mayores de 64 años: Quiromas (vacuna trivalente contra la gripe, adyuvada)

11. ¿Qué vacunas están disponibles en su consultorio / farmacia para administrar a pacientes mayores de 65 años?

- ◯ Solo Vaxigrip Tetra
- ◯ Solo Flucelvax Tetra
- ◯ Solo Chiromas
- ◯ Tanto Vaxigrip Tetra como Flucelvax Tetra
- ◯ Tanto Vaxigrip Tetra como Chiromas
- ◯ Chiromas y Flucelvax Tetra
- ◯ Los tres

PARTE 2 - Preguntas de la encuesta para España – Valencia

12. ¿La preferencia del paciente por una de las vacunas influye en su elección del tipo de vacuna?

- ○ Si
- ○ No

13. ¿La disponibilidad de vacunas condiciona su elección del tipo de vacuna?

- ○ Si
- ○ No

Por favor comente sobre la disponibilidad de vacunas

[ ]

14. ¿Las características individuales del paciente (excepto la contraindicación) influyen en su elección del tipo de vacuna en adultos mayores de 65 años?

- ○ Si
- ○ No

PARTE 2 - Preguntas de la encuesta para España – Valencia

15. ¿Qué características del paciente influyen en su elección del tipo de vacuna (marque todo lo que corresponda)

- [ ] Edad más joven
- [ ] Edad avanzada
- [ ] Presencia de múltiples comorbilidades
- [ ] Presencia de comorbilidades específicas (por favor especificar abajo)
- [ ] Mayor severidad de las comorbilidades
- [ ] Fragilidad
- [ ] Otro, por favor especificar

16. Comente cómo esto afecta su elección del tipo de vacuna (¿es más probable que le indique qué vacuna?)
