# Supplementary material for: Factors driving choices between types and brands of influenza vaccines in general practice in Austria, Italy, Spain and the UK
Source: PLoS One. 2021 Jun 15;16(6):e0252836. doi: 10.1371/journal.pone.0252836 (PMC8205138; doi:10.1371/journal.pone.0252836)
Supplement: Attachment — Submitted filename: Response to Reviewers.docx [file pone.0252836.s003.docx]

*Response to editor and peer-reviewers “Factors driving choices between types and brands of influenza vaccines in general practice in Austria, Italy, Spain and the UK”
April 29, 2021*

| **EDITOR** | **Response** |
| --- | --- |
| 1. Please ensure that your manuscript meets PLOS ONE's style requirements, including those for file naming. |  |
| 2. To comply with PLOS ONE submission guidelines, in your Methods section, please provide additional information regarding your statistical analyses. In addition, please report your p-values to support your claims. | The statistical analysis in this study was very straightforward, only descriptive analyses were performed. In the methods section we have now added that this consists of counts and proportions.    No hypothesis testing or comparisons were done, therefore the use of p-values is not applicable to our study. No claims are made based on the results for any of the products mentioned. |
| 3. In your manuscript you state that "surveys are available in the Supplementary Material." However, we have not received these. Please upload the surveys as supplementary material. | The surveys have been added as supplementary material. |
| 4. Competing interests and funding statements | Please see revised statements in the cover letter |
| 5. Please include captions for your Supporting Information files at the end of your manuscript, and update any in-text citations to match accordingly. | Added. |
| **REVIEWER #1** |  |
| This paper presents the descriptive results of a survey conducted among GP's in four European countries on how they promote influenza vaccination in their clinics and how they choose which influenza vaccine to administer to patients. These findings have implications for vaccine effectiveness estimates, as well as offer insight into vaccine selection when multiple influenza vaccine options are available. The statistical methods used are basic, but I believe they are appropriate. |  |
| **Introduction:** |  |
| nicely written | Thank you. |
| **Methods:** |  |
| - Lines 130-133: The methods do not explain how open-ended responses were analysed and categorised into themes. Please describe what methods were used. Were themes defined a priori? | For the open-ended questions, similar responses were grouped and the number of responses corresponding to each group was quantified. The response groups were defined a posteriori. |
| - It would be interesting to know how the survey questions were developed. Were they derived from previous research or designed specifically for this study? If the latter, was any theoretical framework used in their development? | The questions were designed specifically for this study. No theoretical framework was used, however the survey was pilot-tested in each country prior to use. |
| **Discussion:** |  |
| -The first paragraph is somewhat repetitive at times. For example, lines 291-296 and lines 313-315 present similar information, as well as lines 335-337 and 304-307 . I suggest rewriting the paragraph to make it somewhat more concise. | The discussion has been reorganized to avoid repetition. |
| -Lines 378-380: As you mention, response rates in Italy were quite low (14%). It would be helpful to have some more discussion on how this may have biased results. | Added a sentence on non-response bias. “Non-response bias cannot be excluded; for example GPs participating in the survey could be more interested in influenza which may translate in differences in how they approach the influenza vaccination campaigns.” |
| **REVIEWER #2** |  |
| As an important component to advancing evaluation of effectiveness of influenza vaccines, Anke L et al conducted a descriptive study to describe the factors that influence the choice in use of influenza vaccine types and brands among general practitioners and risk category vaccinees in four European Countries. Annually, several influenza vaccines are recommended in Europe. Factors influencing the use of these vaccines are many and can vary by health providers as well as by those getting vaccinated. The authors conducted a study to better understand these factors in 4 European countries. The research question/s proposed by the authors are of public health importance. Overall, the manuscript is well written. It is easy to read. | Thank you. |
| **Methods:** |  |
| The research methods are clear and straightforward. However, the inclusion of Spain-Valencia is somewhat confusing given it was not participating in the DRIVE project. The choice of this site requires a stronger rationale (Line 94-98). Or was it convenient? | Rephrased. Spain-Valencia is participating in DRIVE. The difference is that in Valencia only one vaccine type was finally recommended in the ≥65y age group in 2019/20 (even though in practice more were used). |
| Information on how the reliability of the survey tool used was assessed would be beneficial to add.? | Reliability: SurveyMonkey is one of the leading online survey platforms for data security and data compliance regulations; and has been successfully used in previous research by some of the co-authors. <https://www.surveymonkey.com/mp/data-security-and-compliance/> |
| Similarly, regarding the online survey tool, information has not been provided on whether participation could occur more than once | The online tool was set to collect responses anonymously and disabling multiple responses from the same device. This has now been added to the methods section. |
| Details on the sample size that was sufficient to answer the study questions are missing. | No sample size calculation was performed. No hypothesis testing was done as the study is purely descriptive. |
| **Results** |  |
| The results are well presented. | Thank you |
| Table 2, lines 145-150: the numbers in parenthesis are not clearly explained and this is somewhat confusing. | This refers to the number of GPs that participated in the survey by region. This has not been added in the footnote. |
| Some results are presented as absolute numbers only; other results are presented as absolute numbers (proportions). It is best to be consistent. | Percentages have been added. |
| A comment on huge difference in the response rates across countries would be useful. | Added. |
| **Discussion** |  |
| The discussion is well written. Limitations and areas for future research are well captured in the manuscript. Is there any bias that could have made the results less reliable? The manuscript is well written with good scientific and public health applications. The authors have done a good work. | Two sentences on non-response bias and on possible hesitancy to admit that GPs have a bias in their vaccine choice has been added to the study limitations. |
